# Iron regulation by Sirtuins 1 & 2 and their therapeutic modulation by Sirtinol

**DOI:** 10.3389/fphar.2026.1882801

**Published:** 2026-08-28

**Authors:** Koffi Amegble, Ludan Liu, Toni Duan, Kyle Fang, Michael S. Petronek, Charvann K. Bailey

**Affiliations:** 1 Department of Biology, Grinnell College, Grinnell, IA, United States; 2 Department of Radiation Oncology, University of Iowa Healthcare Free Radical and Radiation Biology Program, Iowa City, IA, United States

**Keywords:** cancer therapy, iron chelation, oxidative stress, SIRT 1 (sirtuin 1), SIRT 2, sirtinol

## Abstract

Sirtuins 1 & 2 (SIRTs 1 & 2) are deacetylases that are overexpressed in several types of cancer, thereby contributing to various hallmarks of cancer, such as increased proliferation and chemoresistance. Therefore, these enzymes represent potential therapeutic targets. Sirtinol is a small-molecule pharmacological inhibitor of SIRT1 and SIRT2 that shows promise as an anti-tumor drug in several types of cancer. Treatment with Sirtinol reduces tumor proliferation and invasiveness and enhances the cytotoxic effects of other targeted therapies. In addition to its enzyme-inhibitory activity, sirtinol has iron-chelating properties that may contribute to its cytotoxicity in tumor cells by disrupting iron metabolism and inducing oxidative stress. However, the detailed mechanism(s) by which Sirtinol may cause iron-induced oxidative stress to kill tumor cells remain unknown. Furthermore, Sirtinol’s anti-cancer activities and the mechanism(s) by which it induces cytotoxicity have not been extensively evaluated. This review focuses on the following topics: 1) the role of SIRTs 1 & 2 as tumor promoters, providing a rationale for developing inhibitors like sirtinol for these enzymes; 2) examine the connection between SIRTs 1 and 2 and iron metabolism; 3) we examine sirtinol’s anti-tumor properties in relation to its ability to function as a tridentate iron chelator.

## Introduction

Sirtuins 1 & 2 are potential targets for anticancer therapy. Studies show that sirtuins 1 & 2 (SIRTs 1 & 2) are associated with various characteristics of cancer, including chemoresistance and enhanced proliferation (Kojima et al., 2008). Knocking down SIRTs 1 & 2 in cancer cells, by contrast, decreased tumor growth and sensitized several types of cancer to chemo- and radiation-therapy (Wang F, 2012; Portmann et al., 2013). These studies indicate that sirtuins may be a promising target for future cancer treatments.

Sirtinol is a small-molecule pharmacological inhibitor of SIRT1 and SIRT2 and has demonstrated anticancer activity in tumors (Kojima et al., 2008; Jiang et al., 2021). Studies using sirtinol indicate that it promotes oxidative stress in various contexts (Zarzuelo et al., 2013; Mao et al., 2016; Li et al., 2025) and has iron-chelation properties. Its cytotoxic effects may be related to an iron-dependent mechanism (Bayanbold et al., 2023). This proposed mechanism is significant because iron drives required functions such as oxygen transport, energy production, respiration, and DNA synthesis and repair. There is an overaccumulation of iron in cancer cells that propagates redox cycling, resulting in oxidative stress through a Fenton-type reaction which can result in HO• production. These radicals can damage DNA, proteins, and lipids, which can enhance apoptosis or ferroptosis (Dixon et al., 2012; Torti et al., 2018; Petronek et al., 2019). Cancer cells mitigate this oxidative stress by up-regulating antioxidant systems to facilitate cell survival while allowing for iron utilization. Using iron chelators like sirtinol (Gautam et al., 2015; Akam et al., 2016) may offer a novel means of stripping cancer cells of this key metal and, in the process, promote cell death (Bayanbold et al., 2023). We examine the effects of sirtinol, a small pharmacological inhibitor of SIRT1 & SIRT2, as well as the tumorigenic effects of SIRT1 & SIRT2. We propose that Sirtinol may have context-dependent use in cancer therapeutic regimens due to its iron-chelation-based mode of action.

## Sirtuins 1 & 2

Sirtuins were first discovered in *Saccharomyces cerevisiae* as Sir2 (*S*ilent *I*nformation *R*egulator 2) (Rine and Herskowitz, 1987). They are classified as class III lysine deacetylases with a distinct NAD^+^-dependent reaction mechanism. Sirtuins catalyze the deacetylation of lysine residues on target proteins, producing nicotinamide, O-acetyl-ADP-ribose (AADPR), and a deacetylated lysine residue on the target protein (Rine and Herskowitz, 1987; Feldman et al., 2012).

Among the seven sirtuin isoforms identified in humans, SIRT1 and SIRT2 are particularly notable for their roles in regulating cell survival, metabolism, and stress responses. Their activity is linked to the cellular metabolic state, as they function as redox sensors that respond to fluctuations in the NAD^+^/NADH ratio, an indicator of oxidative stress. This NAD^+^-dependent activity connects them to key metabolic pathways such as glycolysis, β-oxidation, and the Krebs cycle, reinforcing their role in cellular energy balance and redox homeostasis (Singh et al., 2018).

Beyond their metabolic functions, SIRT1 and SIRT2 also play important roles in maintaining genomic stability by influencing DNA repair and cell cycle regulation. As tumor suppressors, SIRT1 and SIRT2 contribute to genomic integrity by deacetylating key regulators of DNA repair and mitosis. For example, Sirt2 maintains chromosomal stability during mitosis by positively regulating the anaphase-promoting complex/cyclosome (APC/C). Its loss is associated with increased centrosome amplification, aneuploidy mitotic cell death, and tumor formation; thus connecting SIRT2's role in maintaining genomic integrity to tumorigenesis (Kim et al., 2011).

SIRT1 and SIRT2’s roles in cancer are complex, as they can act as both tumor suppressors and promoters depending on cellular context. They promote tumor progression by modulating oxidative stress responses. Cancer cells exploit Sirt 1-mediated deacetylation of p53 to suppress apoptosis, while SIRT2 deacetylates antioxidant proteins, allowing tumor cells to tolerate elevated levels of reactive oxygen species. Higher expression of these sirtuins has been linked to enhanced tumor proliferation and chemoresistance (Zhou et al., 2024).

SIRT1 and SIRT2’s role as redox-sensitive enzymes makes them critical regulators of the oxidative stress response. While excessive oxidative stress can lead to DNA damage and apoptosis, cancer cells utilize sirtuins to maintain an elevated redox status to drive proliferation without being lethal. Given their roles in redox homeostasis, DNA repair, and tumorigenesis, SIRT1 and SIRT2 remain promising targets for anticancer therapy.

## SIRTs 1 & 2 and cancer

The overexpression of sirtuins has been implicated in cancer progression and therapeutic response. In non-small cell lung cancer (NSCLC), both SIRT 1 and SIRT 2 are upregulated at the protein level in tumor tissues and cancer cell lines compared to normal lung epithelial cells. Elevated expression of either sirtuin is associated with reduced recurrence-free survival. Notably, combined high expression of SIRT1 and SIRT2 identifies patients with significantly poorer recurrence-free and overall survival and serves as an independent prognostic factor after adjustment for tumor stage. Inhibition of SIRT1 and SIRT2 suppresses NSCLC cell proliferation, whereas patients with high SIRT1 expression show reduced responsiveness to chemotherapy (Zhang H, 2013).

SIRT1 also contributes to cancer drug resistance (Wang et al., 2013). SIRT1 enhances chronic myeloid leukemia’s ability to acquire mutations that contribute to BCR-ABL-driven drug resistance via the error-prone DNA damage response pathway (Yamamori et al., 2010). BCR-ABL mutations induce drug-resistance by transcriptionally activating SIRT1. SIRT1 activation deacetylates and activates Ku70, which repairs DNA damage via the error-prone non-homologous end joining (Wang et al., 2013). SIRT1 also activates several other proteins in the DNA damage repair pathway that may contribute to chemotherapeutic resistance, such as apurinic/apyrimidinic endonuclease-1 (APE-1) (Yamamori et al., 2010).

This study showed that SIRT1 deacetylates and activates APE-1 which boosts its DNA repair properties in the presence of genotoxic or oxidative stress. These results suggests that SIRT1 expression correlates with chemotherapeutic resistance.

Furthermore, SIRT1 was significantly elevated in pancreatic cancer cells treated with gemcitabine (Zhang, 2014), a form of chemotherapy. These studies suggest that SIRT1 is involved in an adaptive response to chemotherapy-induced DNA damage and may contribute to chemotherapeutic resistance in cancer. SIRT1 knockdown reduces the proliferation, migration, and invasion of pancreatic cancer cells (Jin et al., 2017). The pancreatic cancer cells showed a 10%–15% reduction in proliferation 72 h after SIRT1 knockdown, along with a 33%–39% decrease in migration. In addition, a 40%–45% reduction in the invasion of the cells was observed. The reduced migratory and invasive capacities induced by SIRT1 knockdown in pancreatic cancer cells were further confirmed by the reduced expression of invasive markers MMP-2, MMP-9 and an increase in E-cadherin (Zhao et al., 2011; Jing et al., 2016). PANC-1 cells treated with SIRT1 shRNA undergo G0/G1 cell cycle arrest and are sensitized to chemotherapies such as 5-FU and gemcitabine. Knockdown of SIRT1 by siRNA also reduced proliferation in adrenocortical tumor cells by 40%–50% and resulted in a significant decrease in cell viability and cell motility (Chimento et al., 2021). This study also reported the same regulation efficacy by a small molecule inhibitor, sirtinol.

Several studies suggest that SIRT2 has malignant properties. Tian et al. found that knockdown of SIRT2, which is highly expressed in osteosarcoma cells, inhibits cancer cell viability and migration (Tian et al., 2022). SIRT2 upregulates the transcription factor Snail, inhibiting its degradation and promoting tumor metastasis. A similar effect was observed for SIRT2 in relation to the PGAM5 malic enzyme 1 pathway (Gongyu, 2023). Here, SIRT2 increased NADPH and lipid synthesis. Lipid accumulation exacerbates the reduction in immunogenic cell death and drives cell-death resistance to 5-fluorouracil and oxaliplatin treatments in liver cancer. SIRT2 also extended SLUG protein lifespan (Zhou et al., 2016), promoting invasive and metastatic features of basal-like breast cancer, known to be one of the most aggressive subtypes of breast cancer. SLUG can also promote cancer cell stemness, which is of key interest given the ability of cancer stem cells (CSCs) to function as a tumor-initiating point.

Wei et al. found that renal cell carcinoma cells with high SIRT2 expression have CSC properties such as elevated CD133, a CSC marker, tumor sphere formation, and resistance to fluorouracil-induced apoptosis (Wei et al., 2018). These correspond with Zhao et al.’s findings that SIRT2 promotes cancer cell stemness and chemoresistance in endometrial cancers through activation of the MEK/ERK signaling pathway (Zhao et al., 2022). These studies, combined, support SIRT2’s role as a tumor promoter and a therapeutic target for overcoming treatment resistance in cancers characterized by SIRT2 upregulation.

However, SIRT2 also has therapeutic properties. SIRT2 also regulates a host of mitotic activities; its loss leads to centrosome amplification, aneuploidy, impaired APC/C activity, and abnormal chromatin modifications, suggesting a more complex role for SIRT2 in cancer. SIRT2 also safeguards genome integrity by deacetylating CDK9 during replication stress. Its loss results in CDK9 inactivation and halts the cell (Zhang T, 2013). In MLL-ENL-driven acute myeloid leukemia (AML), SIRT2 deacetylates lysine 469 on the ENL protein, suppressing cell proliferation but promoting chemotherapy resistance (Hao et al., 2022).

Studies that focused on SIRT2 inhibition reveal a promising future path for cancer treatment. Liu et al. discovered that SIRT2 is upregulated in neuroblastoma cells and in c-Myc-overexpressing pancreatic cancer cells, and that it, in turn, enhances N-Myc and c-Myc protein stability, thereby promoting cancer cell proliferation (Liu et al., 2013). It does this by repressing NEDD4 gene expression by directly binding its promoter. NEDD4 usually binds Myc oncoproteins, targeting them for ubiquitination and degradation. Small-molecule SIRT2 inhibitors restored NEDD4 expression, reduced Myc oncoprotein expression, and suppressed neuroblastoma and pancreatic cancer cell proliferation. Further investigations led to the development of a thiomyristoyl lysine compound, TM, which is a potent SIRT2-specific inhibitor with an IC_50_ of 0.028 μM (Jing et al., 2016). These studies demonstrate the potential of sirtuin inhibitors as anticancer agents in cancer cell lines.

## Connections between SIRTs 1 and 2 & iron metabolism

**FIGURE 1:**
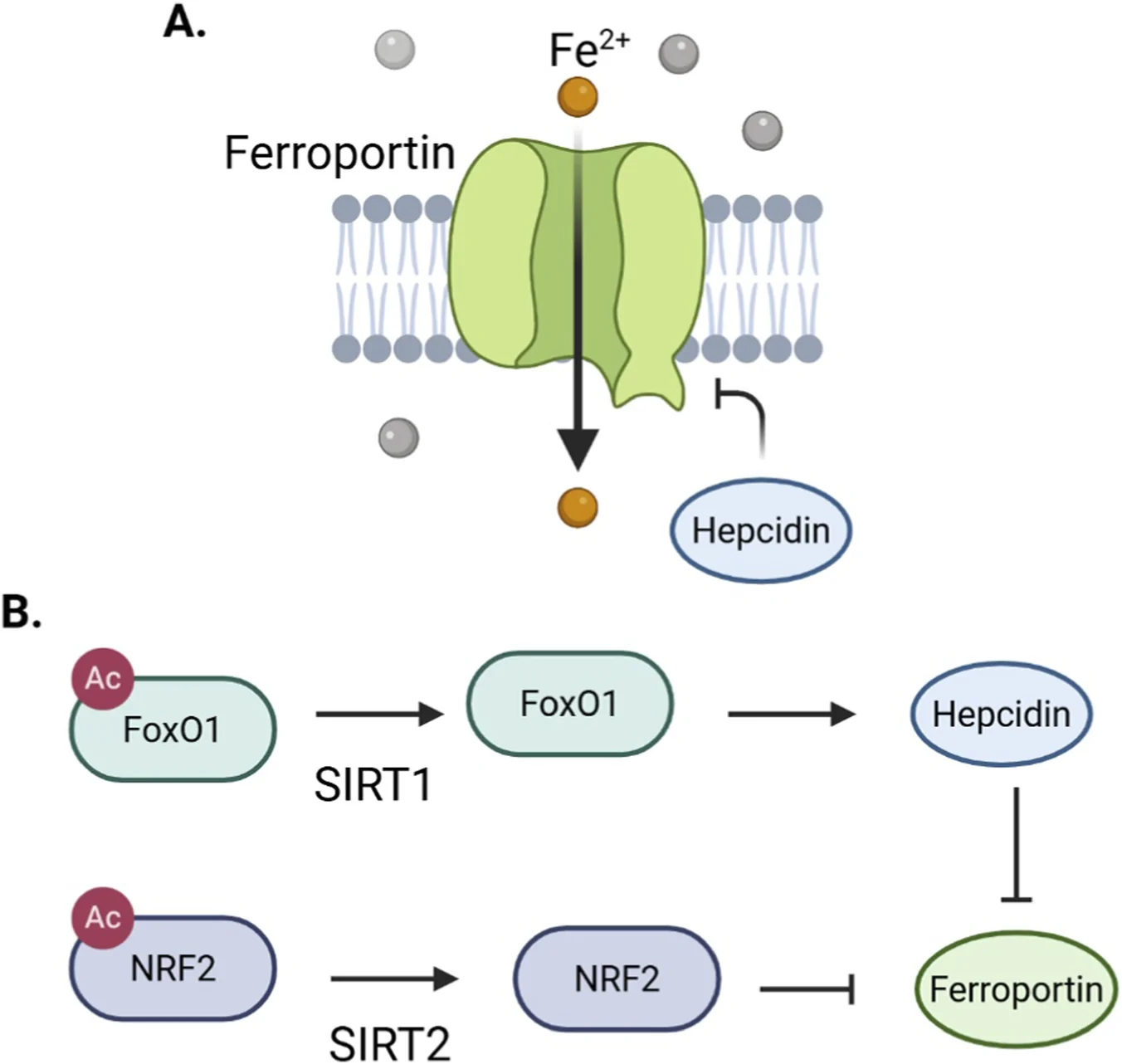
SIRTs 1 & 2 control iron export by regulating ferroportin expression. **(A)** A schematic showing that Iron export by ferroportin is inhibited by hepicidin on the basolateral surface of cells. **(B)** Negative regulation of ferroportin by SIRT1 deacetylation of FOXO1 promotes hepicidin transcription which inhibits ferroportin. SIRT2 deacetylation of NRF2 leads to the transcriptional downregulation of ferroportin. Figure created using BioRender.

Aggressive tumor types frequently exhibit an iron-dependent phenotype (Petronek et al., 2019) yet, molecular connections between oncogenic proteins and iron homeostasis are an active area of exploration. With the direct connections between sirtuins, redox homeostasis, and cancer development it warrants considering how SIRT1/2 may contribute to tumor-associated iron accumulation. Both SIRT1 and SIRT2 have been shown to have direct links to the control of redox active iron levels, primarily in control of iron export through ferroportin (Yang et al., 2017; Ma et al., 2026). SIRT1 can control intracellular iron levels by suppressing iron accumulation through FoxO1-dependent regulation of hepcidin, which is a hormone that binds to the basolateral surface of the cell to promote ferroportin degradation (Figure 1) (Ramey et al., 2010). Xu, et al. recently published a study which showed that the hepcidin gene contains a Foxo binding site and that mice with a FoxO1 deletion exhibit reduced hepatic hepcidin expression accompanied by elevated serum and liver iron content (Xu et al., 2024). Moreover, Li, et al. revealed that SIRT1 deacetylates FoxO1 in pancreatic β-cells to control intracellular iron levels (Li et al., 2025). Conversely, iron overload decreases the expression of SIRT1 resulting in increased FoxO1 acetylation in the nucleus which can be reversed by SIRT1 overexpression (Das et al., 2016), however, the upstream molecular connection between intracellular iron levels and SIRT1-dependent acetylation of FoxO1 have yet to be elucidated. This regulatory feedback may contribute to the tumor promoting effects of SIRT1 as hepcidin has been shown to be secreted in colorectal tumors to support iron-dependent growth (Schwartz et al., 2021).

Sirtuins can also directly control ferroportin expression through NRF2 deacetylation. SIRT1 has been shown to positively regulate NRF2 activity leading to enriched ferroportin expression (Ma et al., 2018). Conversely, a study by Yang et al. showed that SIRT2 directly regulates cellular iron homeostasis by deacetylating and destabilizing the transcription factor NRF2 (Yang et al., 2017) Yang *et al*. Showed that deacetylation by SIRT2 reduces NRF2’s nuclear accumulation and activity, leading to decreased expression of ferroportin (FPN1), the primary iron exporter (Figure 1), and thus increasing intracellular iron levels. SIRT2 deletion in both *in vitro* and *in vivo* models resulted in iron deficiency. Mechanistically, SIRT2 deacetylates NRF2 at lysines 506 and 508 (Yang et al., 2017), which lowers its stability and nuclear localization, suppressing iron export. Furthermore, SIRT2 protects the cells from iron depletion, as SIRT2 knockout in MEFs decreases cell viability. This study also showed that deleting SIRT2 led to a significant increase in transferrin receptor (TfR) and Ferroportin (Fpn1) expression, indicating an iron deficiency response. These data suggests that in the absence of SIRT2, cells attempt to import more iron in response to the cellular iron depletion caused by the increased Fpn 1 expression. Similar to the tumor-promoting effects of hepcidin, Fpn1expression suppresses breast cancer growth (Pinnix et al., 2010).

However, Yang et al. did not measure intracellular iron levels in the absence of SIRT2, leaving it unclear how labile iron was affected. They also found that SIRT2 deletion reduced cell viability under iron-deficient conditions, suggesting that SIRT2 supports cell survival during iron-induced stress. Bayanbold et al. (2023) showed that treatment with sirtinol (a SIRT1/2 inhibitor and iron chelator) caused a dose-dependent increase in TfR and a decrease in ferritin, along with an acute depletion of the labile iron pool in A549 cells, followed by an adaptive rebound over time. Importantly, the toxicity of sirtinol was exacerbated following supplementation with holo-transferrin which suggests that the toxicity may, in fact, be iron-dependent. These findings align with those of Yang et al., as both studies demonstrate that loss or inhibition of SIRT2 activates an iron-deficiency transcriptional program. However, while Yang et al. focused on gene regulation of iron export, Bayanbold et al. (2023) directly measured intracellular iron changes caused by sirtinol. Together, they show that SIRT 2 loss, whether through genetic deletion or chemical inhibition, leads to iron depletion and decreased cell viability, supporting the idea in our study that sirtinol-induced iron loss may contribute to cell death. Taken together, it appears that SIRT1/2 may be critical regulatory features of iron export that can help tumors survive and expand under conditions of high iron dependency.

## Sirtinol and its anti-cancer properties

Sirtinol is one of several small-molecule inhibitors that block the enzymatic functions of SIRT1 and SIRT2. It was discovered and characterized using cell-based screens in yeast *S. cerevisiae* (Bedalov et al., 2001; Grozinger et al., 2001). Further studies showed that sirtinol interacts with both SIRT1 and SIRT2. Sirtinol binds with SIRT1 and SIRT2 via hydrogen bonding interactions via Gln345/His363 and Gln 167, respectively (Peck et al., 2010; Grbesa et al., 2015). Though sirtinol can inhibit both SIRT1 and SIRT2 activity, it prefers to inhibit SIRT1. An *in vitro* study using human recombinant SIRT1 and SIRT2 in the presence of sirtinol and an acetylated substrate confirmed this observation. At identical concentrations of sirtinol, SIRT1 activity is more inhibited than SIRT2 activity. These observations align with the IC50 values of 37.6 uM for SIRT1 and 103.4 uM for SIRT2 (Peck et al., 2010).

Sirtinol’s antitumor properties induce growth arrest in G0/G1 phases of the cell cycle, apoptosis, and reduced cell viability via the Ras–mitogen-activated protein kinase (RAS-MAPK) pathway in non-small cell cancer and breast cancer cells (Ota et al., 2006). Specifically, sirtinol reduced the expression of G0/G1 checkpoint proteins, cyclins B1, D1, and CDK 2 and 6 in both cell types (Wang J, 2012). These observations show that sirtinol reduces proliferation. This may be due to a reduction in proliferating cell nuclear antigen (PCNA) and ribonucleotide hydrolase (Jiang et al., 2021). In MCF-7 and H1299 cells, treatment with 100 uM Sirtinol for 24 h significantly reduced overall cell number and cell viability. Both cancer cell lines displayed increased SA-β-gal activity and expression in a dose-dependent manner—characteristic of senescence-like growth arrest (Ota et al., 2006).

Fong et al. showed that SIRT1 deacetylates FOXO3a, a protein that has proapoptotic activity. Sirtinol causes FOXO3a expression levels to increase and causes the protein to remain in its active, acetylated state (Fong et al., 2014). In adrenocortical carcinoma cells, 40 μM sirtinol induced apoptosis by decreasing the expression of anti-apoptosis protein BCL-2 and increasing the expression of pro-apoptotic proteins BAX and cytochrome c (Chimento et al., 2021). Furthermore, Sirtinol enhances the cytotoxicity of TRAIL-induced apoptosis (Pal et al., 2013). Treatment with sirtinol resulted in growth inhibition and apoptosis in ATL patient cells and other leukemia cell lines (Kozako et al., 2012). Furthermore, in breast cancer cells, sirtinol significantly reduced cancer cell metastasis and vimentin expression and cell motility (Satam et al., 2024).

Jiang et al. showed that Sirtinol decreased the viability and clonogenic survival of esophageal cancer cells (Jiang et al., 2021). Treatment with Sirtinol induced the apoptotic pathway by activating caspase activity while downregulating the anti-apoptotic protein, Bcl-2. Furthermore, proteomic analysis revealed the differential expression of various proteins, which may shed further light on the mechanisms by which Sirtinol induces cytotoxicity in esophageal cancer cells. For example, this analysis revealed a decrease in the replication enzyme, deoxyuridine 5’ triphosphate nucleotidyl hydrolase (dUTPase). Elevated expression of dUTPase is found in several cancers. Several studies suggest that the high expression of dUTPase may result in resistance to 5-FU (Kawahara et al., 2009; Takatori et al., 2010; Wilson et al., 2012).

Furthermore, reducing the expression of dUTPase increases genomic instability in cancers (Chen et al., 2016). DNA damage repair (DDR) is a key feature of genomic stability maintenance, and several studies show that SIRT1 maintains genomic stability by controlling different processes associated with DDR (Jeong and Haigis, 2015; Zhang et al., 2020). Although sirtinol has not been linked to enhancing genomic instability, it is an SIRT1 inhibitor. Therefore, the downregulation of dUTPase by sirtinol may induce genomic instability in esophageal cancers, which may increase their sensitivity to therapies that induce DNA damage.

As outlined above, several studies show that Sirtinol’s potency in cancer cells. However, despite these encouraging results, Sirtinol’s anti-cancer effects are context dependent, and its therapeutic threshold may depend on whether the cancer requires SIRTs 1 and/or 2 for survival. Peck et al. investigated the potency, specificity and cellular targets of three SIRT inhibitors in human breast cancer cells: Sirtinol, EX-527 (a SIRT-1 inhibitor), and Salermide (a SIRT 1 & 2 inhibitor). Proliferation and cell cycle analysis showed that Sirtinol and Salermide, but not EX-527 caused cell death at 50 µM in human breast cancer cells. Furthermore, computational analysis showed that Sirtinol and Salermide, are highly specific for SIRT1 & 2 while EX-527 interacts with SIRT 1, but not SIRT2. *In vitro* SIRT assays using human recombinant SIRT 1 & 2 with a p53 peptide substrate was used to compare the specificity of the three compounds. This experiment showed that although all three compounds are SIRT 1 & 2 inhibitors, EX-527 is highly selective for SIRT 1. Sirtinol and Salermide, but not EX-527 treatment resulted in the *in vivo* acetylation of p53, a SIRT 1 & 2 target and SIRT2 target tubulin (Peck et al., 2010). In contrast, Aimjogjun et al. showed that Sirtinol was the least cytotoxic (IC_50_-73.02 µM) to nasopharyngeal carcinoma cells compared with EX-527 (IC_50_ 5.34 µM) & SIRT2 specific inhibitors AK1(IC_50_: 9.65 µM) and AGK2 (IC_50_ 3.78 µM). Furthermore, SIRT 2 specific inhibitors, AGK1 and AK1 but not sirtinol enhanced the cytotoxicity of lapatinib which is drug commonly used to treat this type of cancer (Aimjongjun et al., 2019).

## Sirtinol as an iron -modulating cancer therapy

Cancer cells typically maintain elevated pools of redox active “labile” iron, which dogmatically serves as a catalyst for oxidative stress (Kruszewski, 2003; Petronek et al., 2019). Labile iron can readily react with H_2_O_2_ through Fenton chemistry to generate damaging hydroxyl radicals or molecular O_2_ to generate O_2_^•-^ (Qian and Buettner, 1999; Nousis et al., 2023). Beyond oxidative stress, the elevation of iron may serve as a critical nutrient to support tumor progression. Iron metabolism serves as a direct link between redox homeostasis and DNA metabolism as iron is readily trafficked through the mitochondria to form iron-sulfur clusters that are inserted into necessary proteins that required for the maintenance of genomic stability (e.g., DNA primase, polymerases, helicases, and glycosylases) (Petronek et al., 2021; Petronek and Allen, 2023). Hence, modulating intracellular iron is a promising type of therapeutic strategy for the control of aggressive tumors.

**FIGURE 2:**
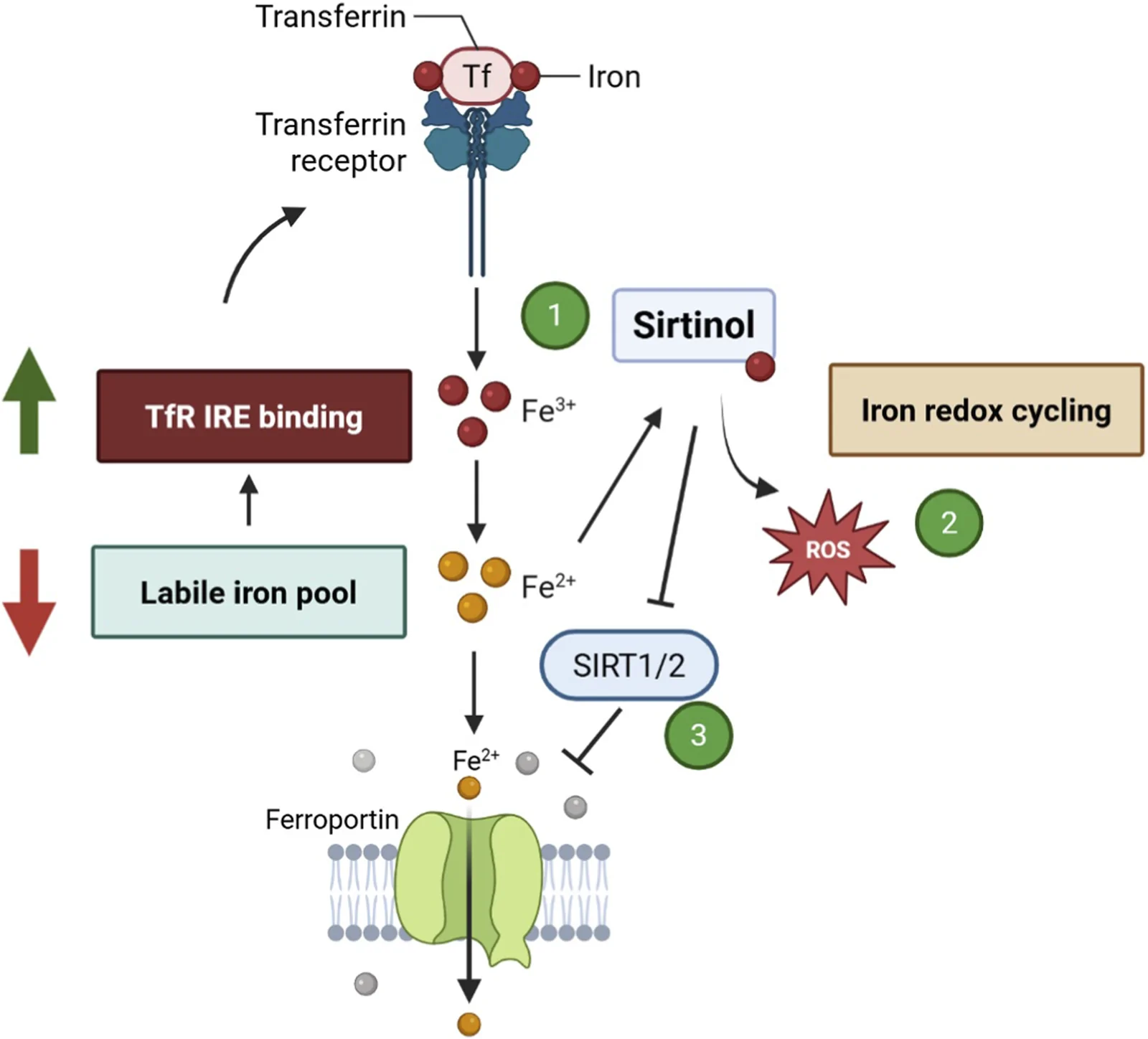
Potential iron-regulatory mechanisms that may lead to Sirtinol-induced ferroptosis. There are three potential iron regulatory connections for sirtinol to induce ferroptosis in cancer cells: 1. Sirtinol chelates and depletes the labile iron pool (red arrow) which results in elevated iron response element binding, TfR protein expression (green arrow), and reflexive iron accumulation; 2. Sirtinol binds intracellular iron and it may facilitate redox cycling to catalyze reactive oxygen species; 3. Under the canonical understanding of sirtinol function, it can inhibit SIRT1/2 resulting in enhanced ferroportin expression, leading to artificially elevated iron export, and a subsequent depletion of the labile iron pool (red arrow) which can trigger detrimental iron fluxes as described in Case 1. Figure created using BioRender.

Although sirtinol is best known as a sirtuin inhibitor, the ability to modulate iron through direct binding is a new facet of sirtinol’s anti-cancer effects that is starting to be explored. Sirtinol contains oxygen and nitrogen ligands capable of coordinating transition metal ions like copper, iron, and zinc (Akam et al., 2016). Interestingly, sirtinol forms stable complexes with both Fe^2+^ and Fe^3+^ *in vitro* and in leukemic cells (Gautam et al., 2015) Atomic iron can coordinate up to six ligands. Therefore, hexadentate iron chelators bind to all six sites of the central iron atom, rendering it inactive (Bogdan et al., 2016; Ibrahim and O’Sullivan, 2020). Tri-dentate chelators, like Sirtinol, occupy three ligand-coordination sites on iron. This leaves several ligand-coordination sites available (Bogdan et al., 2016) on the iron atom, which may make it susceptible to participating in the redox reactions (e.g. Fenton chemistry) that contribute to oxidative stress. While the redox chemistry of sirtinol has yet to be explored, Triapine (a similar tridentate iron chelator and anti-cancer agent) undergoes enzyme-mediated redox cycling to catalyze oxidation reactions. For example, thioredoxin reductase 1 (Trx-1) reduces Fe(III) -Triapine and promotes the redox cycling of iron. This action generates the hydroxyl radical in a peroxide-dependent manner and is thought to contribute to the cytotoxic effects of Fe (III) -triapine (Myers et al., 2013). Consistent with this premise is the study by Akam et al. which showed that pre-formed Sirtinol-iron complexes were more cytotoxic to MCF-7 and MDA-MA-231 breast cancer cells than sirtinol alone (Akam et al., 2016). Furthermore, iron-complexed Sirtinol generated more ROS compared to free sirtinol. The authors suggest that the increased oxidative capacity of the sirtinol-iron complexes may be due to the iron undergoing redox cycling, which leads to oxidative stress linked cell death. Therefore, sirtinol may elicit its cytotoxic effects through the generation of ROS by inducing the redox cycling of iron between its Fe(III) and Fe (II) oxidation states, however, this requires further physiochemical characterization (Figure 2).

Beyond the facilitation of oxidative stress, sirtinol’s chelating properties may simultaneously function to disrupt iron metabolic processes. A recent study showed that in NSCLC cells (A549 & H1299) sirtinol depletes labile iron to a similar degree as deferoxamine, an irreversible iron chelator (Bayanbold et al., 2023). Interestingly, the toxicity of sirtinol was exacerbated in A549 cells which was associated with differential regulation of TfR, suggesting that sirtinol toxicity may be closely tied with tumor-specific iron metabolism. This study revealed that treatment of A549 cells with sirtinol increases and decreases the protein expressions of transferrin receptor (TfR) and Ferritin heavy chain (FtH), respectively. TfR takes up iron into the cell, while Ft-H is part of a protein complex that stores intracellular iron. These data suggests that sirtinol causes an iron deprivation severe enough to elicit a canonical adaptive response (e.g., TfR upregulation and FtH downregulation) to replenish intracellular iron levels. Mechanistically, sirtinol inhibits aconitase activity which supports the hypothesis that it may be stabilizing TfR and repressing FtH translation through IRP1 activation. This adaptive response results in increased levels of redox active iron, indicating that sirtinol may be able to facilitate iron-linked oxidative cell death (i.e., ferroptosis) which warrants further investigation (Figure 2). The iron deprivation effects of sirtinol would be consistent with the previously described induction of apoptosis and G1 cell cycle as iron chelation (e.g., deferoxamine) has been have this same effect in cancer cells (Bajbouj et al., 2018). These findings suggest that sirtinol may not only induce general oxidative stress through sirtuin inhibition but also participate in iron-driven oxidative stress while simultaneously inhibiting iron metabolic function to induce cell death. This dual activity provides a mechanistic link between iron availability, oxidative stress, and sirtinol-mediated cell death which supports expanding the discussion from iron binding to the consideration of sirtinol as an iron-dependent cancer therapy.

## Conclusions and future directions

Due to the importance of sirtuins in the control of tumor redox homeostasis, sirtinol has emerged as a promising candidate for anticancer therapy. Experimental evidence shows that sirtinol disrupts critical pathways regulating tumor proliferation, apoptosis, and metabolic adaptation, yet its full complement of mechanistic properties is still unclear. An emergent mechanism that may play a significant role in its anti-cancer effects is the ability to serve as a tridentate iron chelator. The potential for sirtinol to bind iron while potentially facilitating redox cycling is encouraging for its potential to promote ferroptosis–a well-established cell death mechanism that has yet to be explored in the context of sirtinol toxicity. Moreover, the time-dependent accumulation of labile iron-level through metabolic adaptation may lead to the non-enzyme mediated lipid peroxidation that induces ferroptosis (Figure 2) (Zhang et al., 2025). Although there is no direct link between the sirtuin inhibitory effects of sirtinol and ferroptosis, several studies indicate SIRTs 1 and 2 expression protects cells from ferroptosis which suggest that ferroptosis induction may be a key mechanism of cell death (Zeng et al., 2023). Future studies aimed at evaluating the role of iron modulation in tumors by sirtinol will also need to consider any potential normal tissue toxicities (*e.g.,* liver function) due to the ubiquitous nature of iron metabolism and the emergent evidence of iron accumulation a key component of cancer therapy side effects.

Despite these encouraging findings, current evidence using Sirtinol remains preclinical, with several limitations hindering translation into clinical application. For example, Sirtinol’s effects vary depending on the cell type and cellular context (Hong and Lin, 2021). There are also current preclinical model limitations, since the vast majority of research has been conducted in animal models or *in vitro* models (Hong and Lin, 2021). The dual role of SIRT1 as both a tumor suppressor and tumor promoter, depending on the cancer type, also raises questions about the safety of systemically administering Sirtinol in humans without clear biomarkers (Bosch-Presegue and Vaquero, 2011). Moreover, the optimal dosing strategies and the therapeutic impact of combination approaches remain to be fully elucidated. For example, synergistic interactions with compounds such as valproic acid and dichloroacetic acid highlight the potential of sirtinol to be incorporated into combinations that can amplify cytotoxic effects and overcome resistance mechanisms (Mao et al., 2016).

Future research should prioritize comprehensive pre-clinical *in vivo* studies and well-designed clinical trials to clarify sirtinol’s safety and efficacy profiles. Special attention should be devoted to evaluating its iron-chelating activity, which may represent a novelty for selective tumor targeting. This is because many tumors exhibit heightened iron dependency, representing an intrinsic vulnerability. While sirtinol may further disrupt iron-driven redox and metabolic processes, together, this dual interference with iron biology could enhance tumor responses while limiting effects on normal tissues. Collectively, current evidence supports that sirtinol is a promising anticancer agent, encouraging further investigation to establish its therapeutic potential in clinical settings.
